# Rigid Tissue Increases Cytoplasmic pYAP Expression in Pre-Malignant Stage of Lung Squamous Cell Carcinoma (SCC) In Vivo

**DOI:** 10.3390/cimb44100310

**Published:** 2022-09-29

**Authors:** Muhammad Asyaari Zakaria, May Chee Kiew, Nor Fadilah Rajab, Eng Wee Chua, Siti Fathiah Masre

**Affiliations:** 1 Centre for Toxicology and Health Risk Studies, Faculty of Health Sciences, Universiti Kebangsaan Malaysia, Kuala Lumpur 50300, Malaysia; 2 Centre for Healthy Ageing and Wellness, Faculty of Health Sciences, Universiti Kebangsaan Malaysia, Kuala Lumpur 50300, Malaysia; 3 Faculty of Pharmacy, Universiti Kebangsaan Malaysia, Kuala Lumpur 50300, Malaysia

**Keywords:** lung squamous cell carcinoma (SCC), pre-malignant, phosphorylated Yes-associated protein (pYAP), tissue rigidity, epithelium thickness, cell proliferation, collagen

## Abstract

Increased tissue rigidity is able to activate the Hippo signaling pathway, leading to YAP inactivation by phosphorylation and translocation into the cytoplasm. Accumulating evidence suggests that cytoplasmic pYAP serves as a tumor suppressor and could be a prognostic biomarker for several solid cancers. However, the relationship between tissue rigidity and cytoplasmic pYAP expression in the early stage of lung squamous cell carcinoma (SCC) remains elusive; this was determined in this study by using a mouse model. Female BALB/c mice were assigned into two groups (*n* = 6; the vehicle (VC) and the pre-malignant (PM) group, which received 70% acetone and 0.04 M *N-nitroso-tris-chloroethylurea* (NTCU) for 15 weeks, respectively. In this study, the formation of hyperplasia and metaplasia lesions was found in the PM group, indicating the pre-malignant stage of lung SCC. The pre-malignant tissue appeared to be more rigid as characterized by significantly higher (*p* < 0.05) epithelium thickness, proliferative activity, and collagen content than the VC group. The PM group also had a significantly higher (*p* < 0.05) cytoplasmic pYAP protein expression than the VC group. In conclusion, increased tissue rigidity may contribute to the upregulation of cytoplasmic pYAP expression, which may act as a tumor suppressor in the early stage of lung SCC.

## 1. Introduction

Lung cancer (LC) is one of the leading causes of cancer-related death worldwide. In 2020, LC affected 2.21 million individuals and was responsible for 1.77 million deaths worldwide [1]. According to the Surveillance, Epidemiology, and End Results (SEER) program in 2019, LC’s five-year survival rate remains low at 19.4% [2]. These statistics show that lung cancer is a major public health burden that urgently requires effective therapy. Lung cancer can be divided into two histologic classes: small cell lung cancer (SCLC) and non-small cell lung cancer (NSCLC). NSCLC contributes to >85% of LC cases and can be further classified into adenocarcinoma, lung squamous cell carcinoma (SCC), and large cell lung carcinoma [3]. In the past few decades, the lung SCC subtype has gained attention because its treatment and prognosis remain poorly resolved [4,5]. This unsatisfactory outcome has ensued because lung SCC presents with complex and different genetic alterations compared to other NSCLC subtypes [6,7]. Thus, in depth research to increase our understanding of lung SCC pathogenesis is critical to treat the disease, which is partly clarified in this pre-clinical study.

Recently, the physical properties of cancer tissue, such as its rigidity, have attracted attention. Rigidity has been associated with multiple events that promote cancer growth [8]. Increased tissue rigidity is caused by several factors, mainly by hyperproliferative cancer cells and the oversynthesis of an extracellular matrix protein [9]. Cancer tissue sustains its rigid physical properties by maintaining the positive feedback loop of a complex interaction involving mechanical and oncogenic biochemical cues. In this regard, increased tissue rigidity was found to increase the activity of Yes-associated protein (YAP), which plays a critical role in promoting tumor growth [10,11,12,13]. Active YAP located in the nucleus can stimulate gene transcription involved in cell proliferation and apoptosis suppression by binding to the transcriptional enhancer associate domain (TEAD) [14]. Concurrently, active YAP can stimulate collagen synthesis, collectively establishing a self-reinforcing loop that increases tissue rigidity [15]. In contrast, YAP also can act as a tumor suppressor following its inactivation in a high-cell-density environment, which is one of the characteristics of rigid tissue [16,17]. According to Zhao et al. [16], increased cell density and protein–protein interaction can activate the Hippo pathway, an important signal transduction pathway that regulates tissue homeostasis, which results in YAP inactivation through phosphorylation [18]. YAP is the core downstream effector of Hippo pathway within mammalia, and can be found in both the nucleus and cytoplasm in a normal physiological microenvironment [19,20]. In abnormal rigid tissue, the inactive YAP or phosphorylated YAP (pYAP) is translocated from the nucleus into the cytoplasm, eventually reducing target genes’ transcription activities involving cell proliferation and apoptosis suppression [21,22]. In agreement with the pYAP function, a reduction in murine liver size to normal has been reported if YAP is inactivated, but the progression to hepatocellular carcinoma continues if YAP is activated [23]. Thus, increased cytoplasmic pYAP expression has been suggested as a crucial negative feedback mechanism to reduce carcinogenesis, in which the proliferation and apoptosis equilibrium is disrupted in favor of cancer growth.

Notably, the overexpression of cytoplasmic pYAP protein has been observed in several types of cancer, such as esophageal squamous cell carcinoma [24], breast cancer [25], and colorectal adenocarcinoma [26]. Despite the rising evidence for cytoplasmic pYAP expression in cancer, its expression in lung SCC has not been comprehensively studied. Additionally, the association between tissue rigidity and cytoplasmic pYAP expression in the pre-malignant stage of lung SCC remains to be investigated; this was clarified in this study. Changes in tissue rigidity were attributed to epithelium thickness, proliferation status, and collagen content parameters. At the same time, YAP gene expression and cytoplasmic pYAP protein expression were evaluated using real-time quantitative polymerase chain reaction (RT-qPCR) and immunohistochemistry (IHC) staining, respectively. Finally, we examined the relationship between tissue rigidity parameters such as proliferation status and collagen content and cytoplasmic pYAP expression using the Pearson correlation. 

## 2. Materials and Methods

### 2.1. Animals

Animal ethics approval was obtained from the Universiti Kebangsaan Malaysia Animal Ethical Committee (UKMAEC) (FSK/2017/FATHIAH/24-MAY/846-MAY-2017MAY-2020) and the study was conducted in compliance with the ARRIVE guidelines. Twelve female BALB/c mice or *Mus musculus* (5 weeks; 12–16 g) were purchased from the Animal Unit, Universiti Putra Malaysia. All mice were acclimatized for two weeks with ad libitum access to water and standard mice pallet. All mice were also housed in polypropylene cages containing shredded wood as bedding under the same laboratory conditions of ambient room temperature and lighting (12 h light-dark cycle). After acclimatization for two weeks, the mice were randomly assigned into two groups (*n* = 6): the vehicle (VC) group and the pre-malignant (PM) group. Two days before treatment, the mice were anesthetized intraperitoneally using ketamine and xylazine (KTX) to shave the fur on the dorsal skin for better carcinogen absorption. Mice were topically painted with 25 µL of 70% acetone (ChemAR, Kielce, Poland) and 25 µL of 0.04 M *N-nitroso-tris-chloroethylurea* (NTCU) (Catalog No. N547250; Toronto Research Chemicals, Toronto, Canada) for the VC and PM group, respectively. Treatment was given twice per week with a 3.5-day interval for 15 weeks. Health and behavior assessments were performed on the mice each week in this study. After that the mice were euthanized intraperitoneally by an overdose of KTX followed by cervical dislocation. Then, the lungs were immediately harvested for hematoxylin and eosin (H&E) staining, picrosirius red (PSR) staining, quantitative real-time polymerase chain reaction (RT-qPCR), and immunohistochemistry staining (IHC). The development of pre-malignant lung SCC in vivo in this study was conducted following our previously published work [27]. In our previous work and in this study, we gave 0.04 M NTCU treatment for 15 weeks to induce pre-malignant lung SCC. Then, we confirmed the SCC subtype by the presence of the intracellular bridge and positive expression of cytokeratin 5/6 protein, an SCC biomarker in the malignant stage lung tissues. 

### 2.2. Hematoxylin and Eosin (H&E) Staining

The lung tissues were fixed in 10% formalin (Bendosen, Shah Alam, Malaysia) for 24 h, followed by long-term storage in 70% ethanol at 4 °C. Then, tissues were processed using a tissue processor (Leica TP1020 Leica Biosystem, Wetzlar, Germany) and embedded in paraffin (Thermo Fisher Scientific, Waltham, MA, USA) using a tissue embedder (Leica EG1160 Leica Biosystem, Wetzlar, Germany). In this study, all tissues for histopathology assay were sectioned into 4 µm-thick sections using a microtome (Leica RM2135 Leica Biosystem, Wetzlar, Germany) at the central part of the lung because lung SCC often begins to grow centrally at the main bronchi to bronchioles. The sectioning process was performed perpendicularly to the bronchi divergence and one section was selected consistently from every five sections (~20 μm apart). Then, the tissues were deparaffinized with xylene (Thermo Fisher Scientific, Waltham, MA, USA) and rehydrated in graded alcohol (HmbG Chemicals, Kuala Lumpur, Malaysia). For H&E staining, the tissues were then stained with hematoxylin (Leica Biosystem, Wetzlar, Germany), eosin (R&M Chemicals, Petaling Jaya, Malaysia), and dehydrated with alcohol (HmbG Chemicals, Kuala Lumpur, Malaysia) and xylene (Thermo Fisher Scientific, Waltham, USA) before being mounted with DPX (R & M Chemicals, Petaling Jaya, Malaysia). Tissues were viewed using a light microscope (Nikon Corporation, Tokyo, Japan), and histological scoring was performed and verified by a veterinary pathologist. In this study, three different sections of tissues were examined per mouse. Then, three different airways were randomly analyzed for histological scoring in each section. The scoring was recorded as follows: (a) normal = 0, single-layer columnar epithelium; (b) hyperplasia = 1, more than one layer of the columnar epithelium; (c) metaplasia = 2, a combination of columnar and squamous epithelium while maintaining hyperplastic characteristic; (d) dysplasia = 3, disorientation, presence of cells with high nucleus:cytoplasm, presence of cellular and nuclear polymorphism cells while maintaining hyperplastic and metaplastic characteristics [28]. Subsequently, the mean epithelium thickness was measured from three different sections per mouse using ImageJ 1.46r software (NIH, Bethesda, MD, USA). Then, three different airways were examined in each section. From each airway, three different adjacent areas were analyzed for the measurement of epithelium thickness.

### 2.3. Picrosirius Red Staining (PSR)

Firstly, 4 μm-thick sections were obtained and transferred onto glass slides. After that the tissues were dried at 40 °C overnight. Then, the tissues were deparaffinized and rehydrated in a series of decreasing alcohol concentrations and tap water, before being fixed in Bouin’s solution for one hour at 60 °C. Then, the tissues were stained in PSR (Sigma-Aldrich, Darmstadt, Germany) for one hour. After staining, the tissues were washed with 1% acetic acid (Chemiz, Shah Alam, Malaysia), followed by dehydration with alcohol and xylene before being mounted with DPX [29]. Like the histological scoring, the average percentage of PSR staining was examined from three different sections per mouse. Then, three different airways were randomly analyzed in each section using ImageJ 1.46r software equipped with Sirius Red Macro Plug-in (NIH, Bethesda, MD, USA) [30]. 

### 2.4. Real-Time Quantitative Polymerase Chain Reaction (RT-qPCR)

The lung tissues were homogenized on ice using the ULTRA-TURRAX^®®^ T25 Basic (IKA, Staufen, Germany). Then, the RNA was extracted using InnuPREP Extraction Mini Kit (Analytik Jena, Jena, Germany). The concentration and purity of the RNA samples were measured using an OPTIZENTM NanoQ Microvolume Spectrophotometer (Mecasys Co., Ltd., Daejeon, Korea). Afterwards, good-quality RNA was converted to cDNA using the Tetro cDNA synthesis kit (Bioline, Eveleigh, Australia). In this study, two sets of primer for Yes-associated protein 1 (*YAP1*) and Beta-actin (*ACTB*) genes were used. The primer sequences of *YAP1* or Yes-associated protein 1, forward: 5′-CCCCAGACGACTTCCTCAAC-3′; reverse: 5′-CACATTTGTCCCAGGGAGGG-3′ and *ACTB*, forward: 5′-GTCGAGTCGCGTCCACC-3′; reverse: 5′-GTCGCCCGCGAAGCC-3′ were used. All primers were tested for the optimum annealing temperature to obtain the best universal annealing temperature for all primers. RT-qPCR was performed on a CFX Connect Real-Time PCR Detection System (Bio-rad Laboratories Inc., Hercules, USA) using SensiFAST SYBR No-ROX Kit (Bioline, Eveleigh, Australia) with a three-step thermal cycling protocol. The RT-qPCR starts with an initial polymerase activation at 95 °C for 2 min, followed by 40 cycles of PCR amplification. Each cycle consisted of a 5 s at 95 °C denaturing step, 10 s at 64 °C annealing step, and 20 s at 72 °C extension step. Then, *YAP1* gene expression was normalized with the *ACTB* housekeeping gene and analyzed using the 2^−ΔΔCT^ method [31]. No template control (NTC) and no reverse transcriptase (NRT) controls were included in the RT-qPCR runs.

### 2.5. Immunohistochemistry Staining (IHC)

Firstly, 4 μm-thick sections were obtained and transferred onto charged slides. The sectioned tissues were dried at 40 °C overnight to ensure strong adhesion of the tissues to the slide. The tissues were deparaffinized at 60 °C, followed by immediate transfer into xylene for complete removal of paraffin. After that, the tissues were rehydrated in ethanol and phosphate buffer saline (PBS) before being incubated in 1 × 10 mM sodium citrate buffer (Merck, Darmstadt, Germany) for antigen retrieval. The endogenous peroxidase activity was blocked using 3% H_2_O_2_ (Merck, Darmstadt, Germany) for 10 min, followed by blocking non-specific antibody binding sites using 10% normal goat serum (NGS) (Dako, Glostrup, Denmark) for 10 min. The tissues were incubated with rabbit anti-pYAP monoclonal antibody (Ser127) (diluted to 1:150; Catalog No. 13008; Cell Signaling Technology, Danvers, MA, USA) overnight at 4 °C, followed with anti-rabbit horseradish peroxidase (diluted to 1:100; Catalog No. PI-1000; Vector Laboratories, Newark, NJ, USA) incubation for 1 h at room temperature. After washing with PBS, the peroxidase reactions were developed using the 3,3′-diaminobenzidine (DAB) (Dako, Glostrup, Denmark) for 10 min at room temperature. Counterstaining of the tissues was performed using hematoxylin, followed by tissues’ dehydration in ethanol and xylene before being mounted with DPX. Semi-quantitative analysis of the total pixel intensity of positive DAB brown staining was conducted using ImageJ FIJI 1.52p software (Java 8 version 64-bit) [32].

### 2.6. Statistical Analysis 

All data are presented as mean ± SEM and were analyzed using software SPSS version 25.0 and Graphpad Prism version 8.3.0. The normality test was carried out using Shapiro–Wilk. The differences between groups were statistically analyzed using the student’s *t*-test for the histological score, epithelium thickness, collagen content, and IHC, whereas the correlation between tissue rigidity parameters (Ki67 nuclei protein expression and PSR staining) and cytoplasmic pYAP protein expression was analyzed using the Pearson correlation coefficient. The differences between the groups were considered statistically significant when *p* ≤ 0.05.

## 3. Results

### 3.1. NTCU Induced Pre-Malignant Lesions of Lung SCC In Vivo

**Figure 1:**
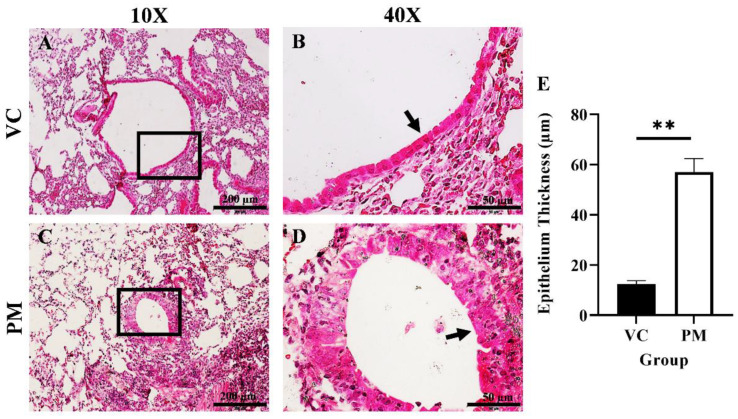
H&E staining of lung tissue from the VC (**A**,**B**) and PM (**C**,**D**) group at 10× and 40× magnification. (**A**) shows normal bronchial epithelial cells and (**B**) shows a single layer of cells contacting the basement membrane, whereas (**C**) shows a thickened bronchial epithelium and (**D**) shows hyperplasia lesions around the bronchial. (**E**) shows the bar chart of the bronchial epithelium thickness (µm) for the VC and PM groups. The arrow points to the epithelial cells. ** in (**E**) indicates *p* < 0.01 using the student’s *t*-test.

The histological changes in the lungs of both the VC and PM group were observed through H&E staining. Figure 1A,B shows a normal single layer of the columnar epithelium at low and high magnification, respectively, for the VC group. Meanwhile, we found hyperplasia and metaplasia lesions in most of the tissues observed for the PM group. Figure 1C,D (the PM group) shows the formation of multiple layers of epithelial cells, namely, hyperplasia (an arrow points to the epithelial cells at the bronchial). We also applied histological scoring, as shown in Table 1. The VC group was found to have an average score of 0, which indicates a normal bronchial epithelium layer. Meanwhile, the PM group has a significantly higher (*p* < 0.05) score of 1.833 ± 0.083, which indicates a mixed formation of hyperplasia and metaplasia lesions. In this study, we also measured the bronchial epithelium thickness in the VC and PM groups. As shown in Figure 1E, the bronchial epithelium in the PM group (57.028 ± 5.389 µm) was significantly thicker (*p* < 0.01) as compared to the VC group (12.493 ± 0.719 µm).

### 3.2. Collagen Content Increased in Pre-Malignant Stage Lung SCC

**Figure 2:**
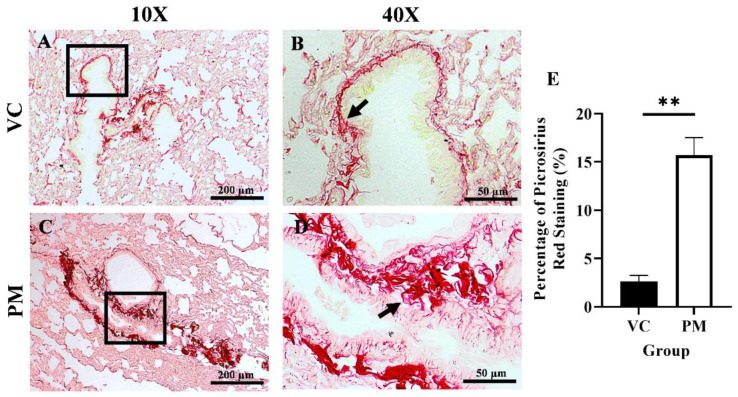
Picrosirius red (PSR) staining for the VC (**A**,**B**) and PM (**C**,**D**) group at 10× and 40× magnification. (**A**) shows PSR staining of normal bronchial area and (**B**) shows observable thin collagen fibrils. (**C**) shows PSR staining of pre-malignant lung SCC at the bronchial area and (**D**) shows an observable thick layer of collagen fibrils. (**E**) shows the bar chart of the percentage of PSR staining of lung tissues from the VC and PM groups. The arrow shows collagen. ** in (**E**) indicates *p* < 0.01 using the student’s *t*-test.

Collagen content in the lungs was observed through PSR staining. Figure 2A,B (the VC group) shows faint red staining with a thin line of collagen fibrils around the bronchial. In contrast, Figure 2C,D (the PM group) show intense red staining with markedly thicker collagen fibrils deposition around the bronchial. Then, we measured the percentage of collagen content as indicated by PSR staining in both groups, and we found that the PM group had a significantly higher collagen content (*p* < 0.01), which was 15.687 ± 1.843% as compared to the VC group (2.623 ± 0.612%) (Figure 2E). 

### 3.3. Proliferation Status Increased in Pre-Malignant Stage Lung SCC

**Figure 3:**
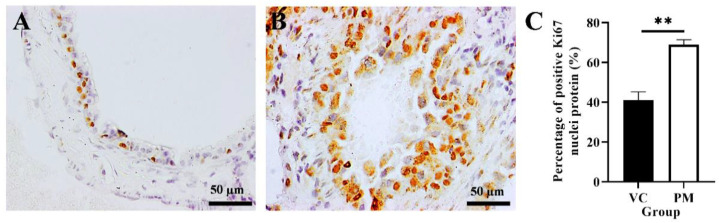
Immunohistochemistry staining from the VC (**A**) and PM (**B**) group for Ki67 protein at 40× magnification. (**A**) shows normal bronchial tissue at 40× magnification with some positive IHC staining of Ki67 nuclei protein. (**B**) shows pre-malignant lung SCC bronchial tissue with a higher positive IHC staining of Ki67 nuclei protein. (**C**) shows the bar chart of the percentage of positive Ki67 nuclei protein (%). ** in (**C**) indicates *p* < 0.01.

The proliferative activity of cells in the VC and PM groups was confirmed by using IHC analysis of the Ki67 protein marker. Figure 3A,B shows that both the VC and PM group have positive Ki67 nuclei protein expression. However, the PM group has a significantly higher percentage of positive Ki67 nuclei (*p* < 0.01), that is, 68.903 ± 2.533% as compared to the VC group (41.043 ± 4.160%) (Figure 3C).

### 3.4. Cytoplasmic pYAP Protein Expression Increased in Pre-Malignant Stage Lung SCC

**Figure 4:**
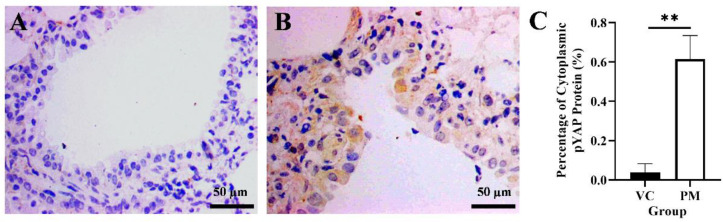
Immunohistochemistry staining from the VC (**A**) and PM (**B**) group for pYAP protein at 40× magnification. (**A**) shows normal bronchial tissue without positive IHC staining of pYAP protein. (**B**) shows pre-malignant lung SCC bronchial tissue with positive IHC staining of pYAP in the cytoplasm. (**C**) shows the bar chart of positive cytoplasmic pYAP protein (%). ** in (**C**) indicates *p* < 0.01.

*YAP1* gene and cytoplasmic pYAP protein expression in lung tissues were identified using qPCR and IHC, respectively. Based on our findings from the qPCR, the PM group was observed to have a higher *YAP1* gene expression with a fold change of 1.805 ± 0.623 as compared to the VC group (1.000 ± 0.020), though no significant difference (*p* > 0.05) was identified between groups. Later, we evaluated pYAP protein expression using IHC and found a positive pYAP protein expression in the cytoplasm from the PM group (Figure 4B). In contrast, no pYAP protein expression was observed in the VC group (Figure 4A). Our IHC findings showed that the PM group had significantly higher cytoplasmic pYAP protein expression (*p* < 0.01), that is, 0.615 ± 0.069% as compared to the VC group (0.037 ± 0.026%) (Figure 4C).

### 3.5. Tissue Rigidity Is Significantly Correlated with Cytoplasmic pYAP Protein Expression

We further analyzed the correlation between cytoplasmic pYAP protein expression and Ki67 protein expression (proliferation status), and collagen content. Notably, we found that cytoplasmic pYAP protein expression was significantly correlated with Ki67 protein expression (r = 0.914, *p* < 0.05) and collagen content (r = 0.973, *p* < 0.05) (Table 2).

## 4. Discussion

The SCC subtype is one of the primary malignancies of lung cancer, and it is characterized by late diagnosis and poor survival rate [33,34,35]. Thus, evaluating the underlying molecular mechanisms of lung SCC is crucial to understand the disease etiology. For the past decades, rigid tumor tissue has been acknowledged as an emerging hallmark of cancer as it plays an essential role in promoting tumor growth [36,37]. However, recent findings also found that increased tissue rigidity can initiate tumor suppressor activity by activating the Hippo signaling pathway, leading to YAP phosphorylation [38,39]. pYAP retained in the cytoplasm is an inactive form of YAP, which is unable to combine with TEAD, thus suppressing the oncogenic events [14]. Therefore, elucidating the mechanistic linkage between tissue rigidity and cytoplasmic pYAP expression is crucial to understand the pathogenesis of lung SCC. 

In this study, we used female BALB/c mice considering the lengthy period of treatment which is 15 weeks. According to An et al. [40], female BALB/c mice were more tolerant and showed less aggressive behavior than males if left in one cage for many weeks. We found that NTCU administration for 15 weeks was able to induce pre-malignant lesions of lung SCC in female BALB/c mice, which was evident by the hyperplasia and metaplasia lesions. In agreement with previous studies, NTCU can induce pre-malignant lung SCC after 8–24 weeks in FVB/n mice [41], which are considered to be in the same category as BALB/c mice in terms of susceptibility to lung SCC induction by NTCU [42]. NTCU is a nicotine derivative, a compound commonly present in cigarettes that can cause cancer by promoting DNA adducts as it is converted into alkylating agent [43]. This carcinogen was chosen because of its high reproducibility and ability to induce lung SCC tissues that harbor a highly similar landscape of mutation to human lung SCC [28,42,44,45,46], making it an excellent model for studying the SCC subtype of lung cancer. 

As cancer progresses, its physical characteristic changes from soft to rigid [47]. This increased tissue rigidity occurs as cancer cells became more proliferative and eventually form a mass of highly dense cells that exerted intracellular force into their surrounding tissues [48,49]. For lung SCC, the cancer cells usually arise from the bronchial epithelium [50]. According to Dacic, assessment of the epithelium is one of the grading criteria required to evaluate the pulmonary lesions [51]. As observed in this study, the thickened bronchial epithelium in the PM group may be due to increased proliferation activity of cancer cells. Herein, we also observed an increase in Ki67 protein in the PM group. Ki67 is a protein that is expressed in all active phases of the cell cycle (G1, S, G2, and M) except in the resting phase (G0), and it is used to mark actively proliferating cells [52]. Our findings are in line with a study conducted by Deneka et al. [53], which found a higher expression of Ki67 in NSCLC than in normal tissues. Besides, increased tissue rigidity is also contributed to by overexpression of ECM components, primarily collagen, which is dysregulated and overproduced in cancer [54,55]. Collagen and laminin are two polymeric networks that make up the basement membrane underlying the bronchial epithelial cells [56,57]. In this regard, we observed a variably thickened basement membrane in the PM group, which may be the result of a significant increase in collagen content. Our finding agrees with previous studies that reported increased collagen content in 3D cultures containing NSCLC cell lines [58] and in various stages of lung SCC tissue obtained from the clinical setting [59].

Hyperproliferative cells and increased collagen deposition are contributors to tensile strength, which increases tissue rigidity. This condition can activate the Hippo signaling pathway, which initiates a cascade that will eventually inactivate YAP by phosphorylation [16]. Through IHC staining, we found higher cytoplasmic pYAP protein expression in the PM group, suggesting an activation of the Hippo signaling pathway in rigid pre-malignant lung SCC tissue. Our finding of increased cytoplasmic pYAP is similar to previous studies conducted by Cao et al. [25] and Yeo et al. [24], which found higher cytoplasmic pYAP expression in an early or lower histological grade of breast cancer and esophageal squamous cell carcinoma (ESCC), respectively. Additionally, increased cytoplasmic pYAP expression was also found in the cancer-associated fibroblast (CAFs) and tumor cells of head and neck squamous cell carcinoma [60]. 

Inactive cytoplasmic pYAP is a key regulator that negatively regulates cancer growth by suppressing cell proliferation and collagen synthesis. However, this study demonstrated a positive correlation between cytoplasmic pYAP protein expression with Ki67. Our finding is consistent with a previous study by Kim et al. [26] that reported a positive correlation between cytoplasmic pYAP with Ki67 expression in colorectal adenocarcinoma. Increased cell proliferation is a common event during an early stage of cancer, as described by Devarakonda and Govindan [61], which might explain the increased Ki67 expression in our pre-malignant lung SCC model. Besides, our study also demonstrated a positive correlation between cytoplasmic pYAP protein expression and collagen content. To the best of our knowledge, this is the first study that shows the correlation between cytoplasmic pYAP and collagen content in cancer. Increased collagen content was believed to inactivate YAP through enhanced crosslinking and collagen binding with transmembrane cell surface receptors [54,62,63]. This collagen–receptor interaction was reported to increase cytoskeleton contractility [64] and inhibit apoptosis [65], which favors cell–cell contact in dense cancer tissue. For example, increased collagen content can activate β1-integrin/focal adhesion kinase receptor [62,64], which then activates the Rho-associated kinase (ROCK) signaling pathway, a cytoskeleton regulator that can enhance cell proliferation, cell migration, cell–cell adhesion, and even promote collagen remodeling and production [9,63,66,67], thereby nurturing a positive feedback loop that increases tissue rigidity, which may lead to Hippo pathway activation and YAP inactivation.

The presence of a positive correlation between cell proliferation and collagen content with cytoplasmic pYAP protein expression may support the idea that rigid tissue can increase cytoplasmic pYAP expression. According to Zhao et al. [16], increased cell density is responsible for the Hippo pathway activation, which leads to YAP inactivation and translocation from the nucleus to the cytoplasm of NIH-3T3 and MCF10A cell lines. Moreover, our finding agrees with a study by Jabbari et al. [68], who reported increased pYAP expression in breast cancer cells cultured in a rigid gel matrix in vitro, suggesting the importance of biophysical cues, especially tissue rigidity, in regulating cytoplasmic pYAP protein expression. Overall, the present study is the first to identify a positive and significant relationship between tissue rigidity and cytoplasmic pYAP in pre-malignant lung SCC in vivo. This could be due to the tissue homeostasis that attenuates lung SCC carcinogenesis. For future studies, the relationship between tissue rigidity and cytoplasmic pYAP at an advanced stage of lung SCC should be investigated to understand the disease. Future work may also look at the YAP and pYAP protein expression using immunofluorescence imaging and Western blot to strengthen the hypothesis. 

## 5. Conclusions

In conclusion, the present study indicated that cytoplasmic pYAP protein expression might be upregulated in response to increased tissue rigidity, suggesting its profound role in suppressing lung SCC growth at an early stage of lung SCC. Factors that contribute to an increase in tissue rigidity, especially increased cell proliferation and collagen may warrant further investigation in the later stage of lung SCC and should be considered as the next therapeutic target to treat the disease in the future.

## Figures and Tables

**Table 1:** The histological score for each group.

Groups	Type of Lesions around Bronchial	Stages of Carcinogenesis	Score (Mean ± SEM)
VC	Normal	No	0
PM	Hyperplasia and metaplasia	Early stage/Pre-malignant	* 1.833 ± 0.083

* *p* < 0.05 using student’s *t*-test.

**Table 2:** Correlation analysis between tissue rigidity parameters and cytoplasmic pYAP expression.

Parameter (%)	Group		Correlation Status
	VC (Mean ± SEM)	PM (Mean ± SEM)	
Cytoplasmic pYAP protein expression	0.037 ± 0.026	0.615 ± 0.069	(r = 0.914, *p* < 0.05)
Ki67 nuclei protein expression	41.043 ± 4.160	68.903 ± 2.533	
Cytoplasmic pYAP protein expression	0.037 ± 0.026	0.615 ± 0.069	(r = 0.973, *p* < 0.05)
Picrosirius red staining	2.623 ± 0.612	15.687 ± 1.843	

*p* < 0.05 using student’s *t*-test.

## Data Availability

All data generated during this study are included in this published article.
